# New Perianal Sepsis Risk Score Predicts Outcome of Elderly Patients with Perianal Abscesses

**DOI:** 10.3390/jcm12165219

**Published:** 2023-08-10

**Authors:** Martin Reichert, Lukas Eckerth, Moritz Fritzenwanker, Can Imirzalioglu, Anca-Laura Amati, Ingolf Askevold, Winfried Padberg, Andreas Hecker, Juliane Liese, Fabienne Bender

**Affiliations:** 1 Department of General, Visceral, Thoracic, Transplant and Pediatric Surgery, University Hospital of Giessen, Rudolf-Buchheim Strasse 7, 35390 Giessen, Germany; l.eckerth@web.de (L.E.); anca-laura.amati@chiru.med.uni-giessen.de (A.-L.A.); ingolf.askevold@chiru.med.uni-giessen.de (I.A.); winfried.padberg@chiru.med.uni-giessen.de (W.P.); andreas.hecker@chiru.med.uni-giessen.de (A.H.); juliane.liese@chiru.med.uni-giessen.de (J.L.); fabienne.bender@chiru.med.uni-giessen.de (F.B.); 2 German Center for Infection Research (DZIF), Site Giessen-Marburg-Langen, Justus-Liebig-University of Giessen, Schubertstrasse 81, 35392 Giessen, Germany; moritz.fritzenwanker@uk-gm.de (M.F.); can.imirzalioglu@mikrobio.med.uni-giessen.de (C.I.); 3 Institute of Medical Microbiology, Justus-Liebig-University of Giessen, Schubertstrasse 81, 35392 Giessen, Germany

**Keywords:** perianal abscess, sepsis, microbiome, elderly, frailty, fistula, emergency surgery, hospital resources

## Abstract

Antibiotic therapy following surgical perianal abscess drainage is debated, but may be necessary for high-risk patients. Frailty has been shown to increase the risk of unfavorable outcomes in elderly surgical patients. This study aims to identify high-risk patients by retrospectively analyzing a single-center cohort and using a pretherapeutic score to predict the need for postoperative antibiotics and extended nursing care following perianal abscess drainage surgery. The perianal sepsis risk score was developed through univariable and multivariable analysis. Internal validation was assessed using the area under receiver-operating characteristic curve. Elderly, especially frail patients exhibited more severe perianal disease, higher frequency of antibiotic therapy, longer hospitalization, poorer clinical outcomes. Multivariable analysis revealed that scores in the 5-item modified frailty index, severity of local infection, and preoperative laboratory markers of infection independently predicted the need for prolonged hospitalization and anti-infective therapy after abscess drainage surgery. These factors were combined into the perianal sepsis risk score, which demonstrated better predictive accuracy for prolonged hospitalization and antibiotic therapy compared with chronological age or frailty status alone. Geriatric assessments are becoming increasingly important in clinical practice. The perianal sepsis risk score identifies high-risk patients before surgery, enabling early initiation of antibiotic therapy and allocation of additional nursing resources.

## 1. Introduction

Perianal abscesses are common diseases in general surgery that require urgent therapy [1]. While simple skin abscesses are usually caused by Staphylococci or Streptococci [2,3,4], the bacteriology, etiology and treatment principles of perianal abscesses are more complex [1]. Perianal abscesses are frequently complicated by fistula-in-ano due to their cryptoglandular origin, which depends on their location [5,6,7,8,9]. Hence, the treatment principles of perianal abscesses are drainage of purulence followed by exploration of potential fistula-in-ano with the consecutive drainage of the fistula, if present, in the emergency setting [6,7,8,9]. The microbiome present in purulence from perianal abscesses is diverse, but specific types of microbiota may indicate certain conditions. For example, the enteric microbiome is predominantly found in abscesses accompanied by fistula-in-ano [1]. Furthermore, certain pre-existing conditions in patients like chronic inflammatory bowel diseases or diabetes can be expected to result in distinct bacteriology [1,10,11,12]. As we have recently shown, the complexity and severity of perianal abscesses including the severity of perineal sepsis are mainly determined by two important factors: the microbial pattern and their acquired drug resistances [1]. While it is still a matter of debate, if antibiotic therapy should be necessary routinely after surgical abscess drainage, it is generally considered appropriate for patients at high risk of extensive perianal infection and consequently poor outcomes [1,13,14,15,16]. Nevertheless, the criteria for postoperative antimicrobial therapy have not been adequately defined.

It is known from other formally trivial diseases in general surgery, that elderly patients are at higher risk for experiencing a more severe and complicated course [17]. However, it is less the chronologic age than the multidimensional frailty syndrome, which dramatically increases the risk for poor outcome and consecutively for additional resource utilization after various elective and urgent surgical interventions [17,18,19,20,21,22]. This is currently unknown for patients with perianal abscesses, thus further research is needed to investigate this relevant issue in more detail. Frailty can simply be measured by the multidimensional modified frailty index (mFi), which shows high predictive values for poor outcome after emergency surgery in elderly patients [17].

The aim of this study was to identify high-risk patients with perianal abscesses who urgently required prolonged nursing resources and additional anti-infective therapy following surgical drainage. Therefore, the study analyzed the relevant risk factors for worse patient outcome and focused on the impact of either older age or frailty on the outcomes of patients with perianal abscesses. This enabled the development of a scoring system which can identify these high-risk patients on admission to the emergency department. With a reliable assessment, the score predicts the need for perioperative antibiotic therapy and additional nursing resources after surgery.

## 2. Materials and Methods

This exploratory, retrospective single-center cohort study was performed in accordance with the latest version of the Declaration of Helsinki and was approved by the local ethics committee of the medical faculty of the University of Giessen (approval No. 66/19). All patients were treated according to the institutional standard-of-care.

From January 2008 to December 2019, all patients (≥12 y of age), who underwent surgical treatment at the University Hospital of Giessen for perianal abscess (i.e., surgical abscess drainage or local abscess excision both with exploration for an accompanying perianal fistula-in-ano and, if present, primary excision or drainage of the fistula) as well as for extended surgical tissue excision for advanced perianal/perineal soft tissue infection originated from perianal abscesses were included in this study.

Patient data were obtained from the prospectively maintained institutional database. Retrospective availability of presented data was >97%. The present work focused on the preoperative frailty status of patients who underwent surgery for perianal abscess. Frailty was assessed independently by two authors of the study in patients ≥60 y of age by using the 5-item and 11-item modified frailty index (mFi-5, mFi-11) [23,24]. Discrepancies were resolved by a third party. Two or more points in mFi-5 and/or ≥3 points in mFi-11 indicated frailty.

C-reactive protein (CRP) values and white blood cell counts (WBC) in peripheral blood were obtained from clinical routine data to assess the extent of systemic inflammation due to perineal infection or sepsis in patients with perianal abscesses. The rates of antibiotic therapy and re-do surgery after the index operation were used to assess the complexity of the disease. Length of hospital stay after index abscess drainage surgery as well as duration until definitive fistula repair or lost in follow-up rate were used as the surrogate parameters for short- as well as long-term outcome, respectively.

Two experienced microbiologists independently reviewed the results of the bacterial cultures and susceptibility tests of swabs obtained from the purulence of perianal abscesses during surgery. The review included assessment of drug resistance of the identified microorganisms based on EUCAST (The European Committee on Antimicrobial Susceptibility Testing) breakpoint tables for interpretation of minimum inhibitory concentrations and zone diameters, v11.0, 2021, as well as intrinsic resistance and unusual phenotypes, v3.2, 2020 (http://www.eucast.org), with focus on acquired drug resistances as described previously [1]. Furthermore, detected isolates were classified according to the ESKAPE definition, which includes highly virulent and frequently drug-resistant pathogens [25].

### 2.1. Surgery and Perioperative Care

The institutional treatment strategies adhere to the German guidelines for anal abscess and cryptoglandular fistula [9]. The standard treatments include emergency surgical drainage or excision of the abscess, followed by careful exploration of the fistula during the index surgery. Primary fistulectomy is typically performed during index surgery for superficial fistulas. In cases of unclear findings or complex fistulas, a temporary loose seton is placed during the index surgery for drainage of the fistula. Fistula repair is then performed after 4–6 weeks, once the infectious situation has been resolved. Surgeons have the discretion to obtain swabs from purulence during abscess-drainage surgery. Thus, swabs are routinely taken from purulence and infected tissue in cases of more severe and complicated perianal disease with extended soft tissue involvement. However, in cases of milder and uncomplicated disease, swabs are not routinely obtained.

Antibiotic therapy is not routinely administered after surgery. Indications for postoperative antibiotic therapy include complicated perianal infection and situations with perianal as well as perineal sepsis with locally advanced phlegmonous or gangrenous soft tissue infection. Postoperatively, patients self-rinse the perianal wounds and are discharged as soon as possible on postoperative day one or two.

### 2.2. Statistical Analyses

The patient cohort was divided into two groups according to age at time of surgery: <60 y and ≥60 y. Subsequently, patients with an age of ≥60 y were subdivided regarding their frailty status to assess the impact of frailty on perioperative patient outcome.

Statistical analyses were performed using GraphPad Prism (Version 9 for Windows, GraphPad Software, San Diego, CA, USA; www.graphpad.com). Two-group comparisons were performed using Fisher’s exact test for categorical data and unpaired, two-tailed Student’s *t*-test for continuous variables. Data are given in *n* (%) or mean ± standard deviation, respectively. If applicable, odds ratios (OR) were calculated using the Baptista–Pike method.

In cases of fistula-in-ano found during index surgery, the duration until definitive fistula repair was calculated by Kaplan–Meier estimation. Patients with an initial drainage of the fistula during index surgery, but were lost in follow-up were censored from this analysis upon the last contact. This is indicated in the Kaplan–Meier curves by vertical ticks. Log rank test was used for Kaplan–Meier curve comparisons.

Spearman’s rho rank correlation was used to determine statistical dependencies between age, frailty, bacteriology and outcome. Results are given as the Spearman’s rank correlation coefficient (r^SP^) and respective significances. Heatmaps display correlation coefficients between the variables.

*p*-Values ≤ 0.05 indicate statistical significance. Because of the exploratory character of the study, no adjustments of *p*-values were performed.

Simple linear regression and multiple linear regression were used for univariable and multivariable analyses, respectively, to evaluate relevant parameters that impact on the outcomes “postoperative antibiotic therapy” and “prolonged length of postoperative hospital stay”. Variables with *p*-values ≤ 0.01 in univariable regression were included in the multivariable analysis. Variables with significance in multiple regression analysis, were included in the perianal sepsis risk score. Areas under the receiver-operating characteristic (ROC) curves were used to estimate the ability of the score, age or frailty alone in predicting the need for postoperative antibiotic therapy or prolonged postoperative hospitalization. To define prolonged hospitalization, we used a threshold of ≥4 d, which exceeded the mean postoperative length of stay of the entire patient cohort (i.e., 3.2 d). To obtain comparability of the ROC curves, areas under the curves (AUC) were directly compared by the method described by Hanley and McNeil [26].

## 3. Results

### 3.1. Patient Characteristics

**Figure 1:**
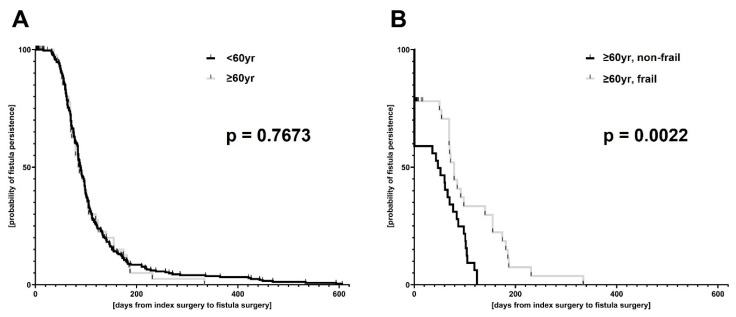
Kaplan–Meier estimation of duration from index surgery to definitive fistula repair. The total patient cohort (**A**) was stratified by age (<60 y versus ≥60 y of age); (**B**) the elderly patient cohort (i.e., patients with an age ≥ 60 y) was subdivided regarding their frailty status (frail versus non-frail).

Overall, 817 patients underwent surgical drainage procedure for perianal abscess during the study period and were included in the data analysis. Of the patients, 693 were younger than 60 y and 124 patients were ≥60 y of age. Older patients suffered more frequently from chronic cardio-pulmonary and metabolic diseases. However, perianal abscesses were rarely associated with chronic inflammatory bowel diseases in the elderly. Although no differences were seen in the rate of fistula-in-ano, perianal disease was more complex in elderly patients, reflected by higher preoperative CRP values, longer operation times as well as higher odds for local severe (gangrenous) tissue infection (OR: 4.281, 95%CI: 1.455–12.680), need for postoperative antibiotic therapy (OR: 1.832, 95%CI: 1.217–2.731) and re-do surgery during short-term follow-up (OR: 2.629, 95%CI: 1.374–4.915). More individuals from the elderly cohort were transferred to the intensive care unit postoperatively and, accordingly, postoperative hospitalization was much longer (Table 1). In this regard, regression analyses reveal discrete linear correlations between patient age, CRP elevation and duration of postoperative hospitalization (Supplementary Figure S1). Nevertheless, no differences were seen in duration until fistula repair nor in overall recurrence rates between younger and older patients (Table 1, Figure 1).

### 3.2. Impact of Frailty on Patient Outcome

From the older patient cohort, 63 patients were classified as being non-frail and 61 as frail. Group comparisons demonstrated that not chronological age alone but rather frailty impacted perioperative outcome. Frail patients suffered from more complex and severe diseases, indicated by higher preoperative CRP and—by tendency—WBC, longer operation times, higher rates of postoperative antibiotic therapy (OR: 4.690, 95%CI: 2.139–10.19), re-do surgery (OR: 7.469, 95%CI: 1.702–34.33) and stool deviation (OR: 8.037, 95%CI: 1.352–91.80) compared with elderly but non-frail patients. Frailty was accompanied with higher odds for postoperative intensive care (OR: 9.358; 95%CI: 1.327–105.4) and prolonged postoperative hospitalization (Table 2). In this regard, especially, the indices from mFi-5 correlated with preoperative markers of systemic inflammation and length of postoperative hospitalization in linear regression analyses (Supplementary Figure S1).

Although advanced age was not, frailty was a potential risk factor for patients to be lost in follow-up for fistula repair by tendency (OR: 3.208, 95%CI: 0.9410–9.808). Furthermore, the duration until fistula repair was significantly longer in frail patients (Table 2, Figure 1).

### 3.3. Bacteriology and Correlation Analyses

Correlation analyses basically confirmed the results of the two-group comparisons. Especially the scores in mFi-5 correlated with relevant outcome data including elevation of preoperative CRP (mFi-5: r^SP^ = 0.211, *p* = 0.020; frailty: r^SP^ = 0.247, *p* = 0.006, age: r^SP^ = 0.110, *p* = 0.002), rates of gangrenous tissue infection (mFi-5: r^SP^ = 0.315, *p* = 0.002; frailty: r^SP^ = 0.232, *p* = 0.010; age: r^SP^ = 0.100, *p* = 0.004), postoperative antibiotic therapy (mFi-5: r^SP^ = 0.355, *p* < 0.0001; frailty: r^SP^ = 0.354, *p* < 0.0001; age: r^SP^ = 0.122, *p* = 0.001), stool deviation (mFi-5: r^SP^ = 0.276, *p* = 0.002; frailty: r^SP^ = 0.205, *p* = 0.019; age: r^SP^ = 0.126, *p* < 0.0001.), re-do surgery (mFi-5: r^SP^ = 0.266, *p* = 0.003; frailty: r^SP^ = 0.265, *p* = 0.003; age: r^SP^ = 0.065, *p* = 0.063), intensive care (mFi-5: r^SP^ = 0.254, *p* = 0.004; frailty: r^SP^ = 0.222, *p* = 0.013; age: r^SP^ = 0.150, *p* < 0.0001), duration of postoperative hospital stay (mFi-5: r^SP^ = 0.442, *p* < 0.0001; frailty: r^SP^ = 0.431, *p* < 0.0001; age: r^SP^ = 0.174, *p* < 0.0001) and the rate of patients who were lost in follow-up for fistula repair (mFi-5: r^SP^ = 0.225, *p* = 0.045; frailty: r^SP^ = 0.212, *p* = 0.059; age: r^SP^ = −0.080, *p* = 0.071).

**Figure 2:**
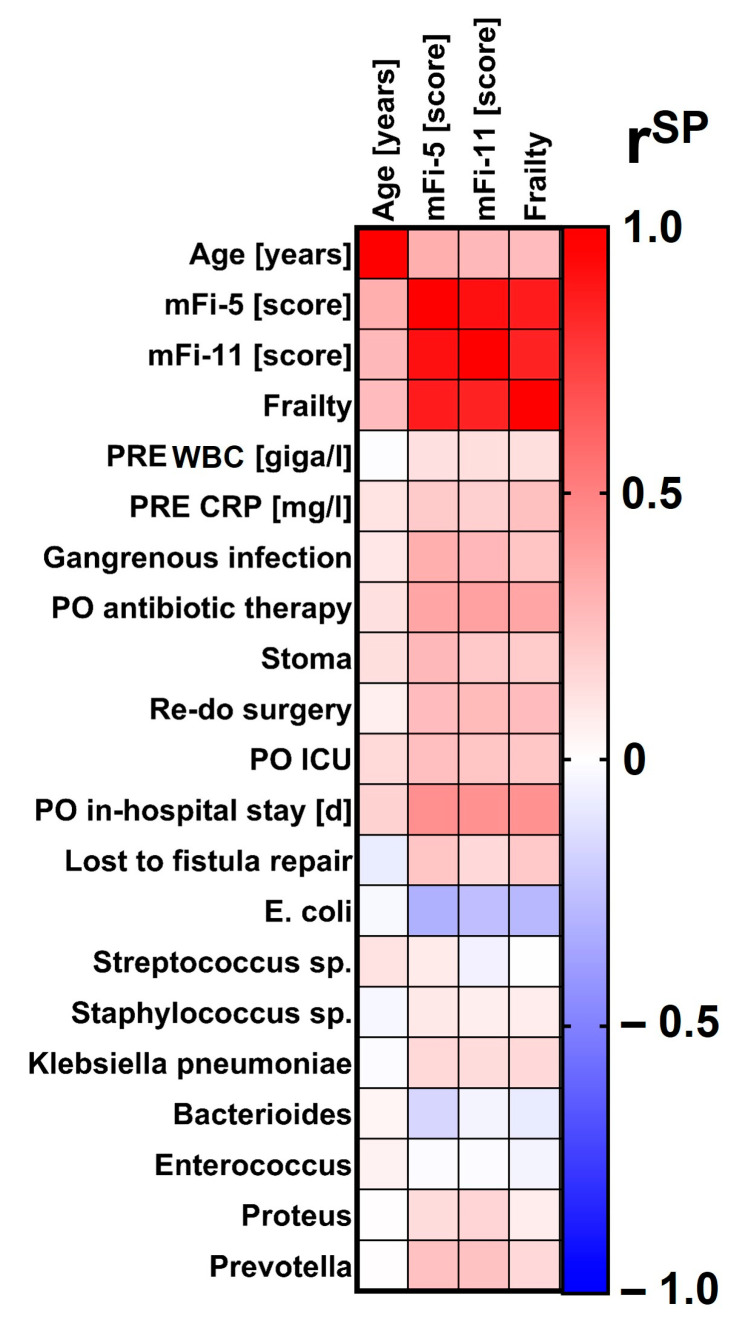
Correlation analysis of chronological age, scores in modified frailty indices or frailty status with relevant perianal disease characteristics. As shown in the legend, the colors code for the Spearman’s rank correlation coefficient (r^SP^). Redder colors indicate the more positive correlation, bluer colors the more negative correlation. mFi-5 = 5-item modified frailty index. mFi-11 = 11-item modified frailty index. PRE = preoperative. WBC = white blood cell count. CRP = C-reactive protein. PO = postoperative. ICU = Intensive Care Unit (including the stay at the intermediate care unit). E. = Escherichia. Sp. = species.

According to the clinical standard [1], overall, 44.2% of the patients received intraoperative swabs from abscesses with a consequent detection of germs in 41.0%. Older age alone but not frailty was associated with higher risk for polybacterial culture. Furthermore, the rate of acquired drug resistances of the detected germs according to EUCAST guidelines was higher in the elderly population. Correlation analyses revealed the detection rate of Streptococcus sp. was higher in elderly patients (mFi-5: r^SP^ = 0.083, *p* = 0.540; frailty: r^SP^ = -0.004, *p* = 0.976; age: r^SP^ = 0.117, *p* = 0.027). The detection rate of E. coli was markedly lower in frail patients; however, age itself did not play a role in that finding (mFi-5: r^SP^ = -0.315, *p* = 0.017; frailty: r^SP^ = −0.285, *p* = 0.032; age: r^SP^ = −0.030, *p* = 0.566). Furthermore, mFi scores but not chronological age correlated by tendency with the finding of Prevotella in perianal abscess swabs (mFi-5: r^SP^ = 0.246, *p* = 0.068; mFi-11: r^SP^ = 0.237, *p* = 0.069; age: r^SP^ = 0.002, *p* = 0.977). No other significant and clinically relevant influences of age or frailty on the specific bacteriology to be expected in perianal abscesses were found in the correlation analyses (Table 1 and Table 2, Figure 2).

### 3.4. Score Development and Validation

Data analysis shows that preoperative scores in mFi-5, CRP, WBC and signs of severe local tissue infection are important factors to consider for postoperative antibiotic therapy and longer hospital stay, i.e., a prolonged need for professional nursing resources (Table 3 and Table 4).

As a result, mFi-5 score, elevated CRP, abnormal WBC and signs of severe local tissue infection were included in the proposed perianal sepsis risk score (Table 5) for perianal abscesses.

The preoperative CRP mean value of the entire patient cohort (49.97 mg/l) was used as the cutoff for a positive score, and each doubling of the value was given an extra point. The sepsis guidelines recommended a preoperative WBC threshold of <4 giga/l and ≥11 giga/L [27].

The score was internally validated using ROC analysis in the retrospective patient cohort, demonstrating a significant predictive power for the need for postoperative antibiotic therapy and prolonged in-hospital stay for perianal sepsis following surgical abscess drainage. This was observed in both the total patient cohort and the elderly patient subgroup (all: *p* < 0.0001).

**Figure 3:**
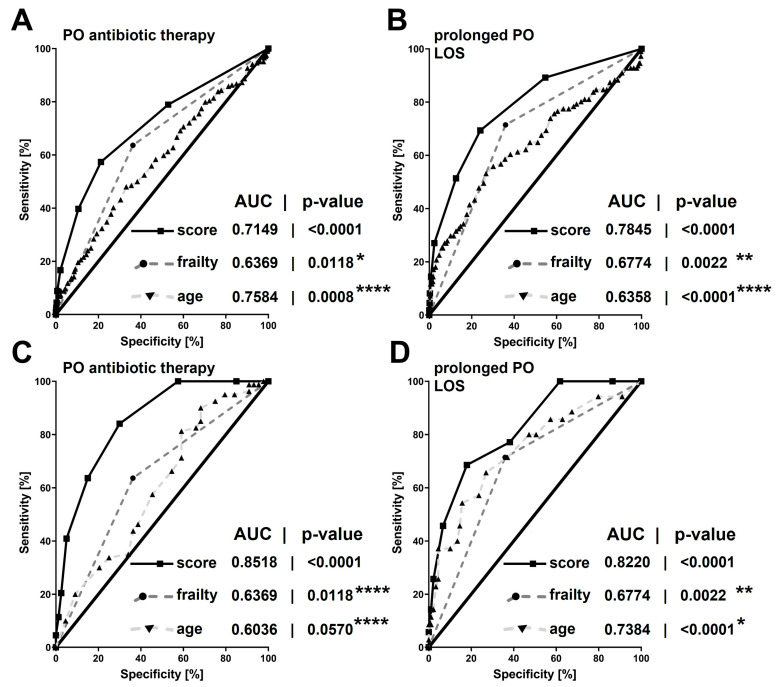
Areas under the receiver-operating characteristic curves of the new perianal sepsis risk score or chronological age and native frailty status alone in predicting the need for postoperative antibiotic therapy (**A**,**C**) or prolonged postoperative hospitalization (**B**,**D**): either in the total patient cohort (**A**,**B**) or in the elderly patient cohort (≥60 y of age; **C**,**D**). Areas under the receiver-operating characteristic curve are given with their respective *p*-values. Asterisks indicate *p*-values obtained from direct comparisons of the areas under the receiver-operating characteristic curves between the new perianal sepsis risk score with native frailty and chronological age alone: * ≤0.1; ** ≤0.05; *** <0.01; **** <0.0001. Four days or more was defined as a prolonged postoperative hospitalization. PO = postoperative. LOS = length of (postoperative) hospital stay. AUC = area under the receiver-operating characteristic curve. Score = new perianal sepsis risk score.

In the direct comparison of AUC by the Hanley and McNeil method [26], the score demonstrated significantly higher predictive values in elderly patients with perianal abscesses compared to the overall patient cohort for both postoperative antibiotic therapy (all patients: AUC = 0.7149 ± 0.0221; 95%CI: 0.6717–0.7582 versus elderly patients: AUC = 0.8518 ± 0.0334; 95%CI: 0.7864–0.9173; *p* < 0.0001) as well as prolonged in-hospital stay (all patients: AUC = 0.7845 ± 0.0246; 95%CI: 0.7364–0.8327 versus elderly patients: AUC = 0.8220 ± 0.0399; 95%CI: 0.7438–0.9001; *p* = 0.0095). Specifically, the predictive value of the score was superior to either age or frailty assessment alone (Figure 3).

In patients with a score of two or higher, there was a higher occurrence of antibiotic therapy and longer hospital stays after surgery. The score was especially effective in elderly patients, with excellent sensitivity at a score of two or higher. The specificity improved either by increasing the score or by adding points from different domains of the score (Table 6).

## 4. Discussion

Our data suggest that the etiology and bacteriology of perianal abscesses in elderly patients differ from the disease in younger patients. However, as is known from other surgical interventions [17,18,19,20,21,22], our data also reveal that the frailty status of aged people—and not their chronological age—mainly determines the outcome in both the short as well as longer term after surgery for perianal abscesses. These findings are the expression of the multidimensional frailty syndrome, which involves physiological and psychological aspects and results in loss of functional reserve and increased vulnerability upon surgery, as stated recently by Cappe et al. [18]. However, not only is the postsurgical situation precarious, because frail patients in our study suffered from more severe and complex diseases (higher rates of severe surrounding tissue infection, higher preoperative CRP values), they also, as a consequence, needed stool deviation, re-do surgery and postoperative antibiotic therapy more frequently.

Routine antibiotic treatment for controlling perianal infection initiated primarily either perioperatively or immediately after surgical abscess drainage with or without approaching fistula-in-ano is disputed in the current literature and certainly not generally necessary for every patient [13,14,15,16,28]. However, our data show that there are patients in whom the well-known principles of abscess therapy “ubi pus, ibi evacua” do not seem to be sufficient alone. Thus, antibiotic therapy in addition to abscess drainage would be beneficial. In this regard, the univariable and multivariable analyses presented here reveal that not only the clinical signs of severe local tissue infection and the laboratory signs of severe systemic inflammatory response but also the frailty status of (aged) patients increase the odds for anti-infective treatment in addition to surgical drainage of perianal abscesses. Furthermore, the sum of these parameters additionally predicts the need for prolonged hospitalization in terms of more intensive treatment and nursing, as well, in these patients. For the sake of simplicity, these parameters either derive preoperatively from clinical routine or can be easily and time-sparingly determined at initial presentation in the emergency room, as is the case for mFi-5 [17]. The parameters were included in the novel perianal sepsis risk score. Thereby, ≥2 points in elderly patients (i.e., ≥60 years of age), best obtained from different domains of the score, were investigated as being highly predictive of the need for both antibiotic therapy and prolonged hospitalization already at the time of first presentation in the emergency room. Hence, the score provides important new insights into treatment modalities of a putative old disease whose therapy is currently debated in an outpatient setting. The score identifies sensitively those patients with high risk for perianal sepsis, poor outcome and situations in which surgery alone is not sufficient for the treatment of perianal abscesses. The perianal sepsis risk score consequently indicates with high sensitivity the need for additional antibiotic therapy and the prolonged need for professional (nursing) care. This correlation analysis presented here as well as the bacteriology and resistance heatmap from the former work by Bender et al. [1] may guide the most effective antibiotic therapy. However, local differences in the resistances profile should be carefully considered.

Our study has impressively shown that frailty status is associated with more complex and severe disease, poorer short-term and long-term outcomes. Despite the high predictive power of the scoring system developed here for the need for antibiotic therapy and prolonged professional (nursing) care in elderly patients with perianal abscesses and perianal sepsis, our study has relevant limitations. The perianal sepsis risk score has been developed and internally validated based on clinical signs of local severe soft tissue infection, laboratory signs of systemic inflammatory response and the frailty status of our cohort of over 800 patients. However, the external validation of the score and thus the evidence to introduce the perianal sepsis risk score into clinical routine is currently pending. This must now be carried out in a future prospective study, so that the score can prove its clinical applicability with its time-efficient calculation as well as diagnostic accuracy. In this prospective setting, it is essential to assess the clinical effectiveness of perioperative antibiotic therapy following abscess drainage, particularly concerning its impact on the outcomes especially of older and frail patients.

## 5. Conclusions

In summary, outpatient care is not favorable for the patients concerned, and the perianal sepsis risk score allows the appropriate measures to be initiated—antibiotic therapy, additional resource calculation and organized ambulatory nursing care—already at the time of initial presentation in the emergency department. The score identifies these high-risk patients, who should be given further optimal access to ambulatory long-term follow-up after acute care.

## Figures and Tables

**Table 1:** Characteristics of patients stratified by age.

Variable	Age < 60 Years *n* = 693	Age ≥ 60 Years *n* = 124	*p*-Value
Female sex, *n* (%)	190 (27.4%)	20 (16.1%)	0.0074
Age, years ± SD	38.9 ± 11.7	67.8 ± 6.1	-
Body mass index, kg/m^2^ ± SD	27.7 ± 7.0	29.2 ± 6.4	0.0246
Diabetes mellitus, *n* (%)	32 (4.6%)	31 (25.0%)	<0.0001
Active smoking, *n* (%)	261 (37.7%)	20 (16.2%)	<0.0001
Chronic pulmonary disease, *n* (%)	43 (6.2%)	19 (15.3%)	0.0013
Coronary artery disease, *n* (%)	12 (1.7%)	18 (14.5%)	<0.0001
CIBD, *n* (%)	73 (10.5%)	5 (4.0%)	0.0201
Systemic immunosuppression ^#^, *n* (%)	32 (4.6%)	10 (8.1%)	0.1211
Duration of surgery, min ± SD	17.33 ± 13.80	22.17 ± 17.81	0.0006
Preoperative laboratory parameters White blood cell count, giga/L ± SD C-reactive protein, mg/L ± SD	11.4 ± 6.1 45.0 ± 61.2	12.1 ± 7.5 77.5 ± 84.4	0.2929 <0.0001
Supralevatoric or pararectal abscess, *n* (%)	25 (3.6%)	4 (3.2%)	1
Gangrenous infection of surrounding tissue, *n* (%)	8 (1.2%)	6 (4.8%)	0.0123
Detection of fistula during index surgery, *n* (%)	426 (61.5%)	80 (64.5%)	0.5481
Primary fistula drainage, *n* (%) Primary fistulectomy, *n* (%)	323 (75.8%) 103 (24.2%)	55 (68.8%) 25 (31.3%)	0.2069
Failure to fistula repair in long-term follow-up, *n* (%)	75 (17.6%)	15 (18.8%)	0.8734
Stool deviation/stoma rate, *n* (%)	9 (1.3%)	8 (6.5%)	0.0017
Postoperative antibiotic therapy, *n* (%) Change in antibiotic therapies, *n* (%)	160 (23.1%) 10 (1.4%)	44 (35.5%) 2 (1.6%)	0.0047 1
Re-do surgery (short-term follow-up), *n* (%)	32 (4.6%)	14 (11.3%)	0.0057
Overall recurrency ^§^, *n* (%)	138 (19.9%)	19 (15.3%)	0.2661
Postoperative stay at intensive or intermediate care unit, *n* (%)	12 (1.7%)	9 (7.2%)	0.0019
Duration of postoperative in-hospital stay, d ± SD	2.9 ± 6.6	5.4 ± 10.0	0.0003
30 day mortality, *n* (%)	1 (0.001%)	1 (0.01%)	0.5889
*n* patients without intraoperative abscess swab *n* patients with intraoperative abscess swab *n* patients without germ detection *n* patients with germ detection	389 (56.1%) 304 (43.9%) 24 (7.9%) 280 (92.1%)	67 (54.0%) 57 (46.0%) 2 (3.5%) 55 (96.5%)	0.6950 0.3996
*n* patients with polybacterial culture ^&^	112 (36.8%)	34 (59.6%)	0.0018
*n* patients with ESKAPE ^$^ bacteria *n* patients with > 1 ESKAPE bacteria	202 (66.4%) 31 (10.2%)	42 (73.7%) 8 (14.0%)	0.3550 0.3608
*n* patients with (acquired) drug-resistant germ(s)	177 (58.2%)	43 (75.4%)	0.0175

^#^ Including chemotherapy within eight weeks before abscess surgery. ^§^ Including patients with recurrent perianal abscess in long-term follow-up after index surgery or perianal abscess in the patient’s history. ^&^ Excluding fungi. ^$^ Although not intended to classify community-acquired infections, the ESKAPE (*Enterococcus faecium*, *Staphylococcus aureus*, *Klebsiella pneumoniae*, *Acinetobacter baumanii*, *Pseudomonas aeruginosa*, *Enterobacter* sp.) definition was used. SD = standard deviation. CIBD = chronic inflammatory bowel disease. CRP = C-reactive protein.

**Table 2:** Characteristics of aged patients stratified by frailty status.

Variable	Age ≥ 60 Years Non-Frail *n* = 63	Age ≥ 60 Years Frail *n* = 61	*p*-Value
Female sex, *n* (%)	13 (20.6%)	7 (11.5%)	0.2230
mFi-5, score ± SD mFi-11, score ± SD	0.5 ± 0.5 0.7 ± 0.7	2.4 ± 0.8 3.1 ± 1.2	- -
Duration of surgery, min ± SD	18.4 ± 12.0	26.6 ± 21.8	0.0123
Preoperative laboratory parameters White blood cell count, giga/L ± SD C-reactive protein, mg/L ± SD	10.9 ± 3.9 58.3 ± 69.6	13.4 ± 9.9 97.9 ± 94.1	0.0625 0.0090
Supralevatoric or pararectal abscess, *n* (%)	2 (3.2%)	2 (3.3%)	1
Gangrenous infection of surrounding tissue, *n* (%)	0	6 (9.8%)	0.0124
Detection of fistula during index surgery, *n* (%)	39 (61.9%)	41 (67.2%)	0.5770
Primary fistula drainage, *n* (%) Primary fistulectomy, *n* (%)	23 (59.0%) 16 (41.0%)	32 (78.0%) 9 (22.0%)	0.0915
Failure to fistula repair in long-term follow-up, *n* (%)	4 (6.3%)	11 (18.0%)	0.0852
Stool deviation/stoma rate, *n* (%)	1 (1.6%)	7 (11.5%)	0.0312
Postoperative antibiotic therapy, *n* (%) Change in antibiotic therapies, *n* (%)	12 (19.0%) 0	32 (52.5%) 2 (3.3%)	0.0001 0.2400
Re-do surgery (short-term follow-up), *n* (%)	2 (3.2%)	12 (19.7%)	0.0042
Overall recurrency ^§^, *n* (%)	7 (11.1%)	12 (19.7%)	0.2185
Postoperative stay at intensive or intermediate care unit, *n* (%)	1 (1.6%)	8 (13.1%)	0.0160
Duration of postoperative in-hospital stay, d ± SD	2.6 ± 2.9	8.4 ± 13.3	0.0009
30 day mortality, *n* (%)	1 (1.6%)	0	1
*n* patients without intraoperative abscess swab *n* patients with intraoperative abscess swab *n* patients without germ detection *n* patients with germ detection	41 (65.1%) 22 (34.9%) 0 22 (100%)	26 (42.6%) 35 (57.4%) 2 (5.7%) 33 (94.3%)	0.0188 0.5175
*n* patients with polybacterial culture ^&^	14 (63.6%)	20 (57.1%)	0.2287
*n* patients with ESKAPE ^$^ bacteria *n* patients with >1 ESKAPE bacteria	19 (86.4%) 4 (6.3%)	23 (65.7%) 4 (6.6%)	0.1242 0.6975
*n* patients with (acquired) drug-resistant germ(s)	16 (72.7%)	27 (77.1%)	0.7584

Two points or more in mFi-5 and/or ≥3 points in mFi-11 indicate frailty. ^§^ Including patients with recurrent perianal abscess in long-term follow-up after index surgery or perianal abscess in the patient’s history. ^&^ Excluding fungi. ^$^ Although not intended to classify community-acquired infections, the ESKAPE (*Enterococcus faecium*, *Staphylococcus aureus*, *Klebsiella pneumoniae*, *Acinetobacter baumanii*, *Pseudomonas aeruginosa*, *Enterobacter* sp.) definition was used. CIBD = chronic inflammatory bowel disease. CRP = C-reactive protein.

**Table 3:** Perioperative determinants for the need for postoperative antibiotic therapy.

Variable	Univariable Analysis		Multivariable Analysis					Included in Score
	r^2^	*p* Value	Coefficients			t	*p* Value	
			B	Std. Error	95% CI			
**Postoperative antibiotic therapy**								
Age [years]	0.016	**0.0003**	0.003	0.007	−0.010–0.017	0.489	0.6260	
mFi-5 [score]	0.125	**<0.0001**	0.094	0.038	0.018–0.170	2.461	**0.0154**	**Yes**
BMI [kg/m^2^]	0.000	0.5257	-	-	-	-	-	
WBC [giga/L]	0.015	**0.0005**	0.001	0.006	-0.010–0.012	0.142	0.8872	
CRP [mg/L]	0.176	**<0.0001**	0.002	0.001	0.001–0.004	4.216	**<0.0001**	**Yes**
Complex abscess ^#^	0.000	0.7036	-	-	-	-	-	
Fistula-in-ano	−0.000	0.8681	-	-	-	-	-	
Fistual seton inserted	0.001	0.3714	-	-	-	-	-	
Gangrenous infection ^§^	0.043	**<0.0001**	0.065	0.205	−0.340–0.471	0.318	0.7507	
CIBD	0.003	0.1308	-	-	-	-	-	
Immunosuppression	0.016	**0.0004**	−0.095	0.138	−0.368–0.177	0.6926	0.4900	
Acquired drug resistances	0.005	0.1845	-	-	-	-	-	
ESKAPE ^&^	0.002	0.4234	-	-	-	-	-	

Perioperative variables that have a significant influence on the need for additional anti-infective therapy in univariable analysis were included in the multivariable regression model. Variables that pass significance in multivariable analysis were included in the new perianal sepsis risk score. ^#^ i.e., Pararectal or supralevatoric abscess. ^§^ Gangrenous infection of surrounding tissue. ^&^ Although not intended to classify community-acquired infections, the ESKAPE (*Enterococcus faecium*, *Staphylococcus aureus*, *Klebsiella pneumoniae*, *Acinetobacter baumanii*, *Pseudomonas aeruginosa*, *Enterobacter* sp.) definition was used. mFi-5 = (preoperative) 5-item modified frailty index. BMI = body mass index. WBC = (preoperative) white blood cell count in peripheral blood. CRP = (preoperative) C-reactive protein value. CIBD = chronic inflammatory bowel disease.

**Table 4:** Perioperative determinants for prolonged hospital stay, i.e., need for prolonged professional nursing resources.

Variable	Univariable Analysis		Multivariable Analysis					Included in Score
	r^2^	*p* Value	Coefficients			t	*p* Value	
			B	Std. Error	95% CI			
**Length of postoperative in-hospital stay**								
Age [years]	0.014	**0.0008**	−0.292	0.191	−0.675–0.091	1.534	0.1317	
mFi-5 [score]	0.144	**<0.0001**	2.714	1.244	0.213–5.216	2.182	**0.0341**	**Yes**
BMI [kg/m^2^]	0.006	0.0238	-	-	-	-	-	
WBC [giga/L]	0.022	**<0.0001**	0.780	0.267	0.243–1.316	2.923	**0.0053**	**Yes**
CRP [mg/L]	0.123	**<0.0001**	0.032	0.016	−0.001–0.064	1.944	0.0577	**Yes**
Complex abscess ^#^	−0.001	0.5172	-	-	-	-	-	
Fistula-in-ano	−0.015	**0.0005**	0.151	2.566	−5.008–5.310	0.059	0.9533	
Fistual seton inserted	−0.005	0.0424	-	-	-	-	-	
Gangrenous infection ^§^	0.318	**<0.0001**	17.460	5.281	6.839–28.080	3.306	**0.0018**	**Yes**
CIBD	0.000	0.9412	-	-	-	-	-	
Immunosuppression	0.003	0.1411	-	-	-	-	-	
Acquired drug resistances	0.023	**0.0037**	3.594	2.860	−2.157–9.345	1.256	0.2150	
ESKAPE ^&^	−0.007	0.1277	-	-	-	-	-	

Perioperative variables that have a significant influence on the length of postoperative in-hospital stay in univariable analysis were included in the multivariable regression model. Variables that pass significance in multivariable analysis were included in the new perianal sepsis risk score. ^#^ i.e., Pararectal or supralevatoric abscess. ^§^ Gangrenous infection of surrounding tissue. ^&^ Although not intended to classify community-acquired infections, the ESKAPE (*Enterococcus faecium*, *Staphylococcus aureus*, *Klebsiella pneumoniae*, *Acinetobacter baumanii*, *Pseudomonas aeruginosa*, *Enterobacter* sp.) definition was used. mFi-5 = (preoperative) 5-item modified frailty index. BMI = body mass index. WBC = (preoperative) white blood cell count in peripheral blood. CRP = (preoperative) C-reactive protein value. CIBD = chronic inflammatory bowel disease.

**Table 5:** Perianal sepsis risk score.

Perianal Sepsis Risk Score [0–9 points]		
Variable	Value	Score
Preoperative **mFi-5** [score]	0 1–5	0 1–5
Preoperative **C-reactive protein** [mg/L]	0–49 ≥50 ≥100	0 1 2
Preoperative **white blood cell count** [giga/L]	≤4 4–10.9 ≥11	1 0 1
Local severe surrounding **tissue reaction** *	No Yes	0 1

* phlegmonous or gangrenous, surrounding the perianal abscess, assessed preoperatively or intraoperatively.

**Table 6:** Perioperative determinants for prolonged hospital stay, i.e., need for prolonged stay.

**Need for PO** **antibiotic therapy**		**Score**	**All Patients (*n* = 817)**				**Elderly Patients (*n* = 124)**			
	**Value**		≥2	≥2 ^#^	≥3	≥3 ^#^	≥2	≥2 ^#^	≥3	≥3 ^#^
	Sensitivity		0.57	0.52	0.40	0.38	1	0.91	0.84	0.77
	Specificity		0.79	0.82	0.89	0.89	0.43	0.59	0.70	0.70
	Positive PV		0.47	0.48	0.56	0.55	0.49	0.55	0.61	0.59
	Negative PV		0.85	0.84	0.82	0.81	1	0.92	0.89	0.85
	Likelihood ratio		2.70	2.82	3.75	3.61	1.74	2.20	2.80	2.58
	Rate of PO antibiotic therapy [*n*] Score < Score ≥		15.3% 47.4% ***	16.4% 48.4% ***	18.3% 55.5% ***	18.7% 54.5% ***	0 48.9 ***	7.8% 54.8% ***	11.1% 60.7% ***	15.2% 58.6% ***
**Prolonged PO hospital stay**	Sensitivity		0.69	0.62	0.51	0.51	1	0.89	0.77	0.74
	Specificity		0.76	0.79	0.87	0.88	0.38	0.53	0.62	0.64
	Positive PV		0.31	0.32	0.39	0.39	0.39	0.43	0.44	0.45
	Negative PV		0.94	0.93	0.92	0.92	1	0.92	0.87	0.86
	Likelihood ratio		2.88	2.93	4.07	4.09	1.62	1.88	2.02	2.07
	Length of PO hospital stay [d] Score < Score ≥		2.3 ± 5.1 5.4 ± 10.3 ***	2.3 ± 5.1 5.7 ± 10.8 ***	2.4 ± 4.8 7.2 ± 13.0 ***	2.4 ± 4.8 7.2 ± 13.1 ***	1.9 ± 0.6 6.7 ± 11.4 *	2.4 ± 2.3 7.5 ± 12.4 **	2.5 ± 1.8 8.5 ± 13.5 **	2.7 ± 2.5 8.5 ± 13.7 **
	Rate of prolonged PO hospital stay [*n*] Score < Score ≥		6.0% 31.2% ***	7.0% 31.5% ***	8.1% 39.0% ***	8.2% 39.2% ***	0 38.9 ***	7.8% 42.5% ***	12.7% 44.3% ***	13.6% 44.8% **

Characteristics of the score in predicting the need for antibiotic therapy in addition to surgical abscess drainage as well as prolonged postoperative length of hospitalization already at first presentation in the emergency department obtained either from the whole patient cohort or exclusively in the subcohort of elderly patients, i.e., ≥60 years of age. ^#^ Points from at least two different domains of the perianal sepsis risk score; *** *p* < 0.0001; **p < 0.01; * *p* < 0.05. PO = postoperative. PV = predictive value.

## Data Availability

The data presented in this study are available on request from the corresponding author.

## Supplementary Materials

The following supporting information can be downloaded at: https://www.mdpi.com/article/10.3390/jcm12165219/s1, Figure S1: Linear relationships between chronological age and modified frailty indices with preoperative markers of systemic inflammation and length of postoperative hospitalization as surrogate parameters for disease severity and short-term outcome, respectively.
