# Supplementary material for: New Perianal Sepsis Risk Score Predicts Outcome of Elderly Patients with Perianal Abscesses
Source: J Clin Med. 2023 Aug 10;12(16):5219. doi: 10.3390/jcm12165219 (PMC10455731; doi:10.3390/jcm12165219)

**Figure S1:** Linear relationships between chronological age and modified frailty indices with preoperative markers of systemic inflammation and length of postoperative hospitalization as surrogate parameters for disease severity and short-term outcome, respectively.

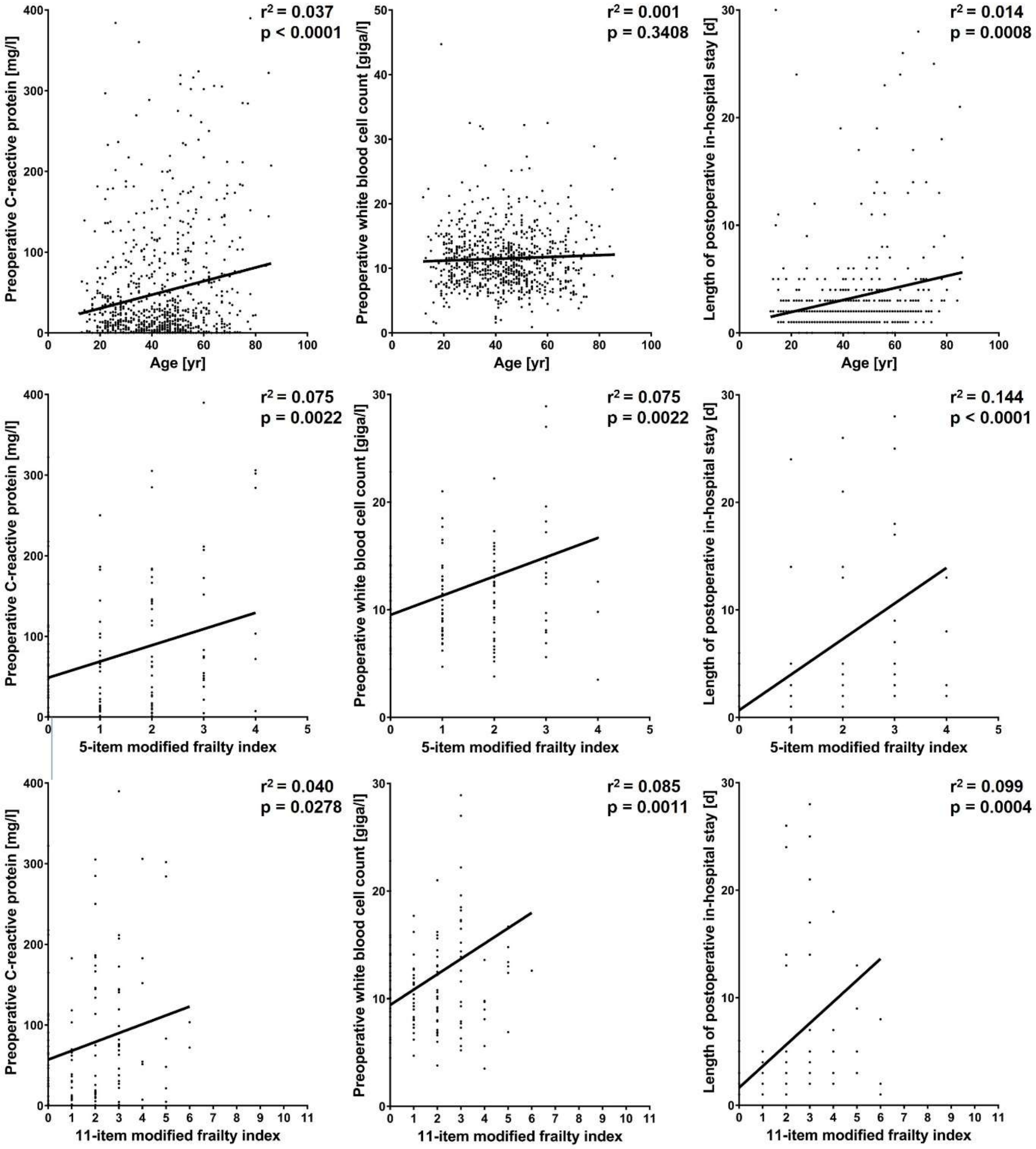

Supplement: Supplementary file 1 [file jcm-12-05219-s001.zip › jcm-2484802-supplementary.pdf]
